# High perioperative lactate levels as a potential predictor for severe acute kidney injury following aortic arch surgery

**DOI:** 10.3389/fmed.2024.1495502

**Published:** 2025-01-06

**Authors:** Dongxu Wang, Chen Yang, Han Wang, Mengmeng Ye, Chao Xue, Weiguang Wang, Bo Yu, Kai Ren, Zhenxiao Jin, Shiqiang Yu, Weixun Duan

**Affiliations:** ^1^Department of Cardiovascular Surgery, Xijing Hospital, Air Force Medical University, Xi’an, China; ^2^Department of Integrated Medicine, Zhongyi-Northeast International Traditional Chinese Medicine Hospital, Shenyang, China; ^3^Department of Cardiovascular Surgery, Shanghai Fourth People’s Hospital, Tongji University, Shanghai, China

**Keywords:** acute kidney injury, acute type A aortic dissection, lactate, mortality, total arch replacement

## Abstract

**Background:**

Acute type A aortic dissection (ATAAD) is a life-threatening condition that often requires total aortic arch replacement (TAR) combined with frozen elephant trunk (FET) implantation. Despite advancements in surgical techniques and preoperative management, postoperative acute kidney injury (AKI) remains a prevalent complication that significantly affects patient prognosis, particularly severe AKI. The aim of this study was to investigate the predictive value of perioperative lactate levels in severe postoperative AKI after TAR.

**Methods:**

A cohort analysis of 328 patients who underwent TAR with frozen elephant trunk implantation at Xijing Hospital, Xi’an, China, between September 2019 and September 2023 was conducted. Patients were categorized according to AKI severity into non-AKI, mild-AKI, and severe-AKI cohorts, and lactate levels were measured at nine perioperative time points. The primary endpoint was severe AKI (Kidney Disease: Improving Global Outcomes stage 3). Uni-and multivariate logistic regression analyses were performed to identify risk factors for severe AKI. Subgroup analysis substantiated the robustness of lactate levels in predicting severe AKI.

**Results:**

In total, 45.4% of patients developed mild AKI postoperatively and 20.7% developed severe AKI. Patients with severe postoperative AKI exhibited higher preoperative lactate levels. Multivariate stepwise backward logistic regression analysis identified lactate levels at 12 h postoperatively ([Lac_po12h_], cutoff value: 3.3 mmol/L; sensitivity: 63.2%; specificity: 72.3%) as an independent predictor of severe AKI. The subgroup analysis underscored the consistent predictive capacity of Lac_po12h_. The 30-day mortality rate was markedly elevated in the severe-AKI cohort, with deceased patients exhibiting a significantly higher Lac_po12h_.

**Conclusion:**

Among patients with acute type A aortic dissection undergoing TAR, high perioperative lactate levels were closely associated with postoperative AKI. Lac_po12h_ is a reliable and effective predictor of severe postoperative AKI, highlighting its clinical utility in risk stratification and management strategies.

## 1 Introduction

In China, total aortic arch replacement (TAR) combined with frozen elephant trunk (FET) implantation has emerged as the standard treatment for patients with acute type A aortic dissection (ATAAD) (1). While the safety profile of this surgical approach has previously been highlighted (2), acute kidney injury (AKI) remains a prevalent postoperative complication of TAR, with an incidence rate ranging from 36 to 66.7% (3). Postoperative AKI is an independent risk factor of mortality and negatively affects patient prognosis following TAR. Severe AKI, defined as Kidney Disease: Improving Global Outcomes (KDIGO) stage 3, significantly affects survival (4). A prospective observational study aimed at developing a robust and efficient method for screening patients at high risk of AKI following ATAAD highlighted patient lactate levels as a potential biomarker (5). Furthermore, prehospital blood pressure and lactate levels have been investigated as early predictors of AKI in patients with trauma (6). Using a database at our institution, this cohort analysis study aimed to investigate the predictive value of perioperative lactate levels for severe AKI following TAR combined with FET implantation in patients with ATAAD, thereby facilitating the strategic utilization of the lactate level as a predictive biomarker for identifying high-risk patients and ultimately enhancing surgical outcomes.

## 2 Materials and methods

This study was approved by the Ethics Committee of Xijing Hospital (KY20192066-F-1) and was registered at the Clinical Trial Registry (NCT03607786) (7). The study adhered to the ethical principles outlined in the Declaration of Helsinki. All patients provided written informed consent.

### 2.1 Patient data

Data regarding the surgical outcomes of 345 consecutive patients diagnosed with ATAAD who had undergone TAR combined with FET implantation at Xijing Hospital between September 2019 and September 2023 were analyzed. Computed tomography angiography findings were used to confirm the diagnosis of ATAAD. Seventeen patients with preoperative end-stage renal disease who required dialysis or who were lost to follow-up for the primary endpoints were excluded from the study. The final study cohort comprised 328 patients who were classified into the following cohorts based on AKI severity: non-AKI, mild AKI, and severe AKI.

### 2.2 Surgical procedure

All patients underwent TAR with FET implantation. This complex procedure involved the use of a four-branch arch Gelweave graft (Vascutek Terumo Inc., Scotland, United Kingdom) for total arch replacement and implantation of a FET device (MicroPort Medical, Shanghai, China) (Supplementary Figure S1) in the descending aorta. Anesthetic induction was commenced using intravenous administration of sufentanil, rocuronium, propofol, and midazolam. Perioperative maintenance of anesthesia was facilitated using a combination of sufentanil, pipecuronium, and midazolam. Cardiopulmonary bypass (CPB) was initiated with cannulation of the brachiocephalic trunk and right atrium, accompanied by left ventricular venting. Systemic perfusion was maintained at a flow rate of 2.2–2.4 L/min/m^2^. Myocardial protection was achieved through antegrade cold del-Nido cardioplegia administration via the coronary orifices following aortotomy. Once the aortic root procedures were completed, moderate hypothermic circulatory arrest ([MHCA], bladder temperature: 25–28°C), and selective antegrade cerebral perfusion (rate, 5–10 mL/kg/min) were routinely applied via the innominate artery and/or the left common carotid artery, and FET and TAR were initiated. Upon completion of the anastomosis, lower body perfusion resumed through the perfusion limb of the four-branch prosthetic graft. Subsequently, the left common carotid artery, proximal aortic stump, left subclavian artery, and iliac artery were sequentially anastomosed to the graft. Rewarming was initiated, and CPB was discontinued. Postoperative analgesia was administered using sufentanil and remifentanil.

### 2.3 Definition and data collection

The primary outcome was the development of postoperative severe AKI (KDIGO stage 3). Postoperative AKI was defined in accordance with KDIGO guidelines (8). Mild AKI (KDIGO stage 1 or 2) was defined as follows: an increase in the serum creatinine level of ≥26.5 μmol/L within 48 h; or a 1.5-to 2.9-fold elevation from baseline and severe AKI with the level exceeding a 3-fold baseline or ≥ 353.6 μmol/L within 7 days; or the requirement for renal replacement therapy. Myocardial injury was defined as cardiac troponin I (cTnI) of ≥0.1 ng/mL. Hepatic dysfunction was defined as aspartate aminotransferase (AST) or alanine aminotransferase (ALT) levels of >40 IU/L. Renal dysfunction was defined as an endogenous creatinine clearance rate of <85 mL/min. The creatinine clearance rate was estimated using serum creatinine (sCr) values derived from the following Cockcroft–Gault formula:


$$\begin{array}{l} \frac{\left[\left(140-\mathrm{age}\right)\times \mathrm{weight}\;\left(\mathrm{kg}\right)\right]}{\left[72\times sCr\;\left(\frac{\mathrm{mg}}{\mathrm{ml}}\right)\right]}for\;\mathrm{males};\\ \frac{\left[\left(140-\mathrm{age}\right)\times \mathrm{weight}\;\left(\mathrm{kg}\right)\right]}{\left[85\times sCr\;\left(\frac{\mathrm{mg}}{\mathrm{ml}}\right)\right]}\;for\;\mathrm{females} \end{array}$$

Paraparesis was defined using the Tarlov score, based on a unilateral or bilateral lower limb muscle strength score of ≤4 combined with iliac artery involvement, as indicated on computed tomography angiography. According to KDIGO guidelines regarding time window restrictions for AKI diagnosis, serum lactate values were collected at nine time points, namely, admission to the operating room, prior to MHCA, at the 5th minute after reinitiation of lower body perfusion, on completion of rewarming, at the end of CPB, at the end of the surgery, and at 4 h, 12 h, and 24 h postoperatively to describe perioperative lactate levels from admission to 24 h postoperatively. Lactate levels at the respective time points were denoted by Lac_adm_, Lac_ca_, Lac_op_, Lacrw, Lac_cpb_, Lac_end_, Lac_po4h_, Lac_po12h,_ and Lac_po24h_. Characteristics denoted with the subscript ‘po12h’ present the levels at the postoperative 12th hour. Clinical data were extracted from hospital databases.

### 2.4 Statistical analysis

Continuous variables of normal distribution were described by the mean value (±standard deviation), tested by one-way analysis of variance, and compared between cohorts by the least-significant difference method or the Games–Howell method. The continuous variables with non-normal distribution were described by the median (25% quartile; 75% quartile). The Kruskal–Wallis H-test was used for comparisons between multiple cohorts, and the Bonferroni correction was applied. The categorical variables were described by frequency n (%), and the x test and the Bonferroni correction were used to make comparisons among multiple cohorts. To determine the sensitivity and specificity of perioperative lactate levels for predicting severe postoperative AKI, receiver operating characteristic (ROC) curve analysis was utilized to identify the optimal time point to measure lactate levels for severe postoperative AKI. Statistical significance was set at *p* < 0.05. Variables exhibiting significant differences (*p* < 0.05) underwent a univariate logistic regression analysis. Subsequently, only variables that maintained statistical significance and passed collinearity diagnostic tests were included in the stepwise multivariate logistic regression analysis. Given the constraints of sample size, multivariate logistic regression was conducted using a stepwise backward elimination method. This approach ensures that only variables with significant associations are included in the model, thereby enhancing the precision and interpretability of the analysis. Subgroup and interaction analyses were performed using univariate and multivariate logistic regression analyses, respectively. Subgroup-specific analysis was stratified according to age, sex, estimated glomerular filtration rate (eGFR), left ventricular ejection fraction (LVEF), and cTnI level to determine the robustness of lactate levels in predicting severe postoperative AKI across diverse demographic and clinical subgroups. All statistical analyses were conducted using SPSS (version 22.0; SPSS Inc., Chicago, IL, United States) software.

## 3 Results

### 3.1 Patient characteristics

The baseline characteristics and surgical details of all the cohorts are presented in Tables 1, 2. Postoperatively, 111 (45.4%) patients developed mild AKI, and 68 (20.7%) developed severe AKI, among whom 48 underwent renal replacement therapy. Moreover, 60% of patients were diagnosed at local hospitals and subsequently transferred to our institution. Influenced by the transfer time, 92 patients (28.0%) underwent surgery <24 h post-onset, 154 (47.0%) underwent surgery between 24 and 48 h post-onset, 36 (11.0%) underwent surgery between 48 and 72 h post-onset, and 46 (14.0%) underwent surgery >72 h post-onset.

**Table 1:** Preoperative characteristics of patients according to AKI severity.

Characteristic	All patients (*n* = 328)	Non-AKI (*n* = 111)	Mild AKI (*n* = 149)	Severe AKI (*n* = 68)	*p*-value	
					Severe AKI vs. non-AKI	Severe AKI vs. mild-AKI
Demographics						
Male	264 (80.5)	91 (82.0)	123 (82.6)	50 (73.5)	0.18	0.125
Age (years)	49.76 ± 9.28	49.12 ± 9.47	50.60 ± 9.18	48.99 ± 9.17	0.927	0.232
BMI (kg/m^2^)	25.08 (22.92,27.68)	24.80 (22.64,27.55)	24.80 (22.93,27.19)	25.99 (23.88,29.39)	0.005	0.009
Comorbidities						
Hypertension (%)	225 (68.6)	74 (66.7)	100 (67.1)	51 (75.0)	0.238	0.241
Myocardial injury (%)	96 (29.3)	30 (27.0)	39 (26.2)	27 (39.7)	0.077	0.044
Pneumonia (%)	10 (3.0)	2 (1.8)	4 (2.7)	4 (5.9)	0.141	0.246
TIA (%)	23 (7.0)	9 (8.1)	10 (6.7)	4 (5.9)	0.578	0.818
Hepatic dysfunction (%)	80 (24.4)	22 (19.8)	32 (21.5)	26 (38.2)	0.007	0.01
Renal dysfunction (%)	110 (33.5)	43 (38.7)	40 (26.8)	27 (39.7)	0.898	0.057
Paraparesis (%)	29 (8.8)	6 (5.4)	12 (8.1)	11 (16.2)	0.017	0.071
Imaging data						
LVEF (%)	56.48 ± 3.74	56.81 ± 3.42	56.41 ± 4.04	56.07 ± 3.55	0.169	0.557
Moderate–severe AR (%)	132 (40.2)	49 (44.1)	53 (35.6)	30 (44.1)	0.997	0.229
Branch artery involvement						
BCT (%)	214 (65.2)	69 (62.2)	104 (69.8)	41 (60.3)	0.803	0.168
LCA (%)	141 (43.0)	47 (42.3)	69 (46.3)	25 (36.8)	0.46	0.188
LSA (%)	141 (43.0)	48 (43.2)	63 (42.3)	30 (44.1)	0.909	0.8
CA (%)	79 (24.1)	20 (18.0)	36 (24.2)	23 (33.8)	0.016	0.138
CT (%)	147 (44.8)	51 (45.9)	63 (42.3)	33 (48.5)	0.737	0.39
MA (%)	99 (30.2)	33 (29.7)	45 (30.2)	21 (30.9)	0.87	0.919
only-LRA (%)	123 (37.5)	50 (45.0)	45 (30.2)	28 (41.2)	0.612	0.112
only-RRA (%)	62 (18.9)	24 (21.6)	31 (20.8)	7 (10.3)	0.052	0.059
Bilateral-RA (%)	27 (8.2)	9 (8.1)	9 (6.0)	9 (13.2)	0.268	0.075
Only-LIA (%)	67 (20.4)	23 (20.7)	33 (22.1)	11 (16.2)	0.452	0.31
Only-RIA (%)	35 (10.7)	15 (13.5)	12 (8.1)	8 (11.8)	0.734	0.381
Bilateral-IA (%)	93 (28.4)	33 (29.7)	40 (26.8)	20 (29.4)	0.964	0.695
Laboratory data						
Hb (g/L)	136.0 (125.0,146.8)	136.1 (125.2,146.5)	136.0 (123.0,147.0)	135.5 (127.0,145.8)	0.686	0.731
WBC (10^9^/L)	12.08 (9.72,14.18)	11.19 (9.12,13.75)	12.12 (9.98,14.16)	13.14 (10.53,16.26)	0.001	0.013
NE (10^9^/L)	9.96 (7.71,12.42)	9.19 (7.09,11.60)	10.19 (8.15,12.73)	11.24 (8.66,14.21)	0.001	0.022
CRP (mg/L)	5.65 (2.23,9.65)	3.74 (1.81,9.54)	5.61 (2.37,9.57)	7.25 (3.18,11.17)	0.134	0.068
AST (IU/L)	25.0 (18.0,36.0)	23.0 (17.0,35.0)	24.0 (17.0,32.0)	31.0 (19.2,43.5)	0.016	0.003
ALT (IU/L)	28.0 (22.0,36.0)	26.0 (21.0,36.0)	27.0 (21.5,35.0)	34.0 (26.2,44.0)	<0.001	<0.001
Glucose (mmol/L)	6.92 (5.98,8.03)	6.83 (5.87,8.16)	6.87 (5.87,7.78)	7.41 (6.55,8.35)	0.33	0.009
Cr (μmoI/L)	76.0 (63.0,96.0)	79.0 (65.0,107.0)	72.0 (61.0,87.0)	83.0 (68.0,127.7)	0.144	0.001
CysC (mg/L)	1.04 (0.91,1.21)	1.03 (0.91,1.25)	1.02 (0.89,1.13)	1.11 (0.94,1.36)	0.053	0.001
eGFR (mL/min/1.73m^2^)	98.3 (73.9,107.8)	94.7 (67.5,105.6)	102.1 (85.1,109.5)	94.2 (55.5,105.3)	0.249	0.001
cTnI (ng/mL)	0.018 (0.005,0.130)	0.010 (0.005,0.108)	0.018 (0.005,0.123)	0.045 (0.010,0.194)	0.016	0.019
BNP (pg/mL)	282.0 (128.3,610.3)	259.0 (94.0,658.0)	265.0 (140.5,556.5)	333.0 (172.7,772.7)	0.2	0.102
PT (s)	11.4 (10.8,12.0)	11.6 (10.9,12.1)	11.4 (10.8,11.9)	11.2 (10.8,11.9)	0.73	0.513

AKI, acute kidney injury; ALT, alanine aminotransferase; AR, aortic regurgitation; AS, aspartate aminotransferase; BCT, brachiocephalic trunk; BMI, body mass index; CA, coronary artery; CRP, C-reactive protein; CT, celiac trunk; cTnI, cardiac troponin I; CysC, cystatin C; eGFR, estimated glomerular filtration rate; Hb, hemoglobin; IA, iliac artery; KDIGO, Kidney Disease: Improving Global Outcomes; LCA, left common carotid artery; LIA, left iliac artery; LRA, left renal artery; LSA, left subclavian artery; LVEF, left ventricular ejection fraction; MA, mesenteric artery; NE, neutrophilic granulocyte; NT-proBNP, N-terminal pro-B-type natriuretic peptide; PT, prothrombin time; RRA, right renal artery; RA, renal artery; RIA, right iliac artery; sCr, serum creatinine; WBC, white blood cell. Non-AKI, no AKI postoperatively; mild-AKI, KDIGO stage 1 or 2 postoperatively; severe AKI, KDIGO stage 3 postoperatively. Severe-AKI vs. non-AKI: patients with severe AKI (KDIGO stage 3) compared with patients without AKI. Severe AKI vs. mild-AKI: Patients with severe AKI (KDIGO stage 3) compared with patients with mild-AKI (KDIGO stages 1 or 2).

**Table 2:** Intraoperative characteristics of patients according to AKI severity.

Characteristic	All patients (*n* = 328)	Non-AKI (*n* = 111)	Mild AKI (*n* = 149)	Severe AKI (*n* = 68)	*p*- value	
					Severe AKI vs. non-AKI	Severe AKI vs. mild AKI
MHCA duration (min)	35.0 (32.0,40.0)	36.0 (32.0,43.0)	34.0 (30.0,39.0)	36.0 (32.0,41.5)	0.683	0.231
MHCA temperature (°C)	26.0 (25.6,26.5)	26.0 (25.6,26.4)	26.0 (25.6,26.5)	26.0 (25.5,26.5)	0.484	0.828
ACC duration (min)	106.0 (93.0,122.0)	105.0 (91.0,117.0)	106.0 (92.5,122.0)	108.5 (96.2,125.7)	0.170	0.128
CPB duration (min)	218.0 (197.0,240.8)	206.0 (194.0,231.0)	291.0 (199.0,237.5)	231.0 (205.5,261.5)	<0.001	0.008
Operation duration (h)	6.58 (5.92,7.42)	6.33 (5.75,7.17)	6.50 (6.00,7.37)	7.33 (6.44,8.00)	<0.001	<0.001
Combined with CABG (%)	22 (6.7)	4 (3.6)	8 (5.4)	10 (14.7)	0.007	0.021
RBC transfusion (U)	3.0 (0,4.8)	2.0 (0,4.0)	3.0 (1.3,4.5)	4.0 (2.0,4.4)	0.149	0.989

ACC, aortic cross-clamping; AKI, acute kidney injury; CPB, cardiopulmonary bypass; CABG, coronary artery bypass grafting; KDIGO, Kidney Disease: Improving Global Outcomes; MHCA, moderate hypothermic circulatory arrest; RBC, red blood cell. Non-AKI, no AKI postoperatively; mild-AKI, KDIGO stage 1 or 2 postoperatively; severe AKI, KDIGO stage 3 postoperatively. Severe AKI vs. non-AKI, patients with severe AKI (KDIGO stage 3) compared with patients without AKI. Severe AKI vs. mild AKI, patients with severe AKI (KDIGO stage 3) compared with patients with mild AKI (KDIGO stage 1 or 2).

Compared with the non-AKI and mild-AKI cohorts, patients in the severe-AKI cohort had a higher preoperative body mass index, white blood cell count, and neutrophil granulocyte (NE), AST, ALT, and cTnI levels, along with higher incidence of hepatic dysfunction (*p* < 0.05). Furthermore, these patients underwent longer CPB and surgery durations and more complex surgical procedures with coronary artery bypass grafting (*p* < 0.05). Postoperative outcomes in the severe-AKI cohort were also more complex, with longer ICU stays and higher incidences of cardiovascular, respiratory, and neurological complications and hepatic dysfunction. The corresponding laboratory results at 12 h postoperatively supported this conclusion (*p* < 0.05), with higher AST, ALT, sCr, cystatin C, and cTnI levels, a lower eGFR, and a longer prothrombin time (PT) in the severe-AKI cohort (*p* < 0.05). The 30-day mortality rate in the severe-AKI group was significantly higher than that in the other two cohorts (*p* < 0.05, Table 3).

**Table 3:** Patient postoperative characteristics according to AKI severity.

Characteristic	All patients (*n* = 328)	Non-AKI (*n* = 111)	Mild AKI (*n* = 149)	Severe AKI (*n* = 68)	*P*-value	
					Severe AKI vs. non-AKI	Severe AKI vs. mild AKI
ICU LOS (d)	4.0 (3.0,6.0)	4.0 (3.0,5.0)	4.0 (3.0,5.5)	7.5 (5.0,12.0)	<0.001	<0.001
Cardiovascular complications (%)	87 (26.5)	25 (22.5)	31 (20.8)	31 (45.6)	0.001	<0.001
Respiratory failure (%)	91 (27.7)	21 (18.9)	35 (23.5)	35 (51.5)	<0.001	<0.001
Pneumonia (%)	67 (20.4)	11 (9.9)	23 (15.4)	33 (48.5)	<0.001	<0.001
Stroke (%)	17 (5.2)	4 (3.6)	4 (2.7)	9 (13.2)	0.016	0.002
Delirium (%)	59 (18.0)	14 (12.6)	23 (15.4)	22 (32.4)	0.001	0.004
Hepatic dysfunction (%)	100 (30.5)	16 (14.4)	44 (29.5)	40 (58.8)	<0.001	<0.001
30-day mortality (%)	40 (12.2)	3 (2.7)	4 (2.7)	33 (48.5)	<0.001	<0.001
Laboratory data						
Hb_po12h_ (g/L)	112.0 (102.0,123.0)	116.6 (106.2,126.2)	112.0 (98.0,123.0)	107.0 (98.0,117.5)	0.007	0.183
WBC_po12h_ (10^9^/L)	10.33 (8.42,12.83)	10.54 (8.36,12.69)	9.84 (8.19,12.29)	10.73 (8.57,13.67)	0.425	0.151
NE_po12h_ (10^9^/L)	8.83 (7.00,10.09)	9.05 (6.81,10.52)	8.45 (6.81,10.59)	9.45 (7.73,11.92)	0.244	0.109
AST_po12h_ (IU/L)	29.5 (21.2,50.0)	28.0 (21.0,39.0)	28.0 (20.0,46.5)	43.0 (25.0,109.0)	<0.001	<0.001
ALT_po12h_ (IU/L)	79.0 (58.0,124.0)	72.0 (56.0,96.0)	77.0 (55.5,122.5)	117.0 (73.2,257.2)	<0.001	<0.001
sCr_po12h_ (μmoI/L)	110.5 (86.0,146.7)	92.0 (76.0,114.0)	113.0 (88.0,143.0)	153.0 (125.2,209.7)	<0.001	<0.001
CysC_po12h_ (mg/L)	1.12 (0.93,1.42)	0.98 (0.82,1.15)	1.15 (0.96,1.41)	1.48 (1.13,2.00)	<0.001	<0.001
eGFR_po12h_ (mL/min/1.73m^2^)	64.81 (44.81,86.68)	82.37 (61.19,97.33)	63.57 (46.83,82.16)	40.67 (29.45,55.42)	<0.001	<0.001
cTnI_po12h_ (ng/mL)	5.39 (2.77,10.92)	4.28 (2.50,8.02)	5.64 (2.67,11.01)	7.47 (3.80,16.77)	<0.001	0.021
NT-proBNP_po12h_ (pg/mL)	530.5 (247.7,1109.5)	452.0 (178.0,908.0)	544.0 (283.5,1198.5)	642.5 (360.2,1636.5)	0.006	0.183
PT_po12h_ (s)	13.4 (12.5,14.3)	13.1 (12.2,14.1)	13.3 (12.4,14.1)	13.9 (12.8,15.6)	<0.001	0.002

AKI, acute kidney injury; ALT, alanine aminotransferase; AST, aspartate aminotransferase; cTnI, cardiac troponin I; CysC, cystatin C; eGFR, estimated glomerular filtration rate; Hb, hemoglobin; ICU LOS, intensive care unit length of stay; KDIGO, Kidney Disease: Improving Global Outcomes; NE, neutrophil granulocyte; NT-pro BNP, N-terminal pro-B-type natriuretic peptide; PT, prothrombin time; sCr, serum creatinine; WBC, white blood cell. Non-AKI, no AKI postoperatively; mild-AKI, KDIGO stage 1 or 2 postoperatively; severe AKI, KDIGO stage 3 postoperatively; po12h, level at the respective indicator at 12 h postoperatively. Severe AKI vs. non-AKI, patients with severe AKI (KDIGO stage 3) compared with patients without AKI. Severe AKI vs. mild AKI, patients with severe AKI (KDIGO stage 3) compared with patients with mild-AKI (KDIGO stage 1 or 2).

### 3.2 Association between perioperative lactate levels and severe AKI

**Figure 1:**
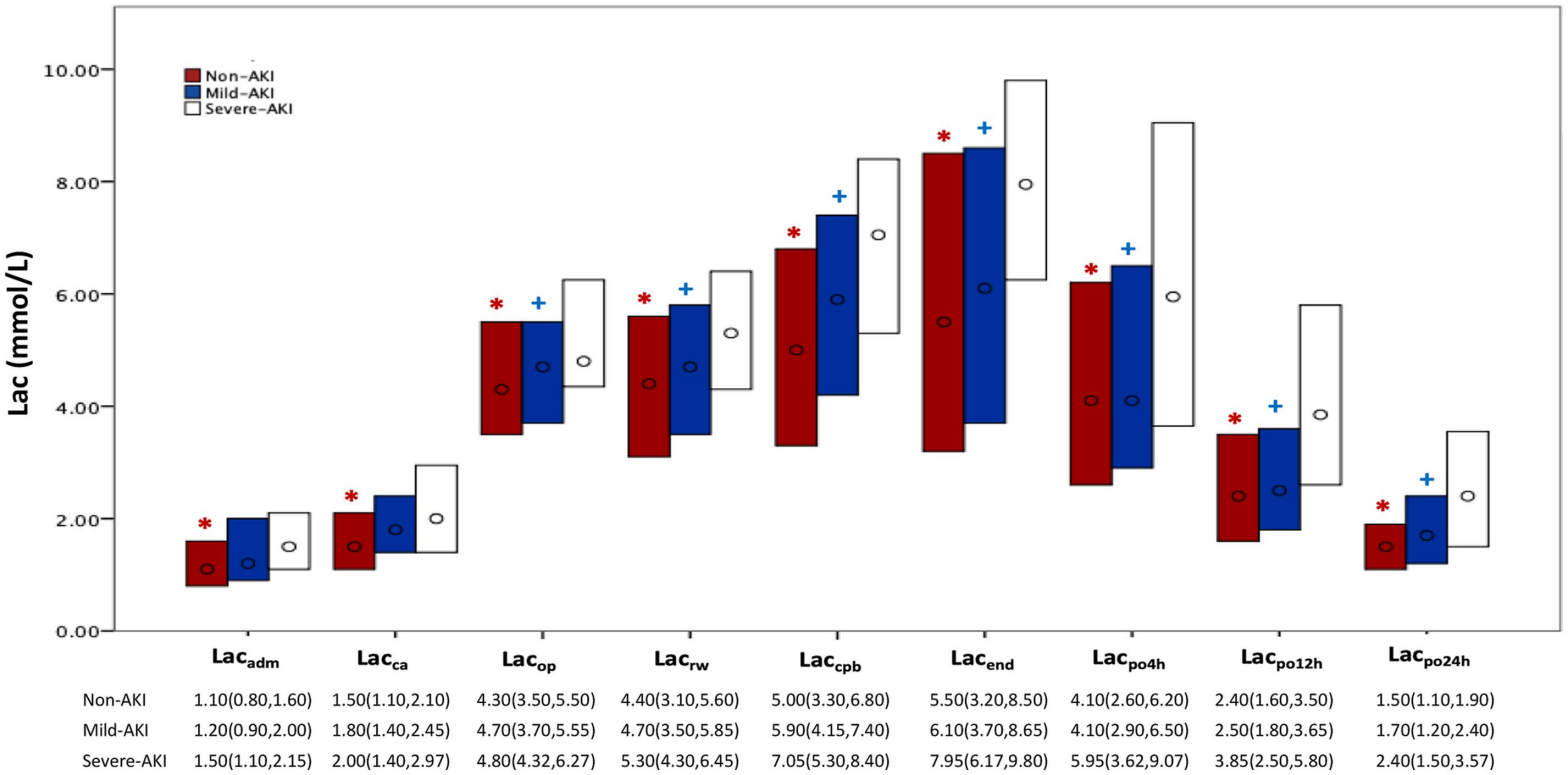
Perioperative lactate levels according to AKI severity. AKI, acute kidney injury; Lac, lactate; non-AKI, no AKI postoperatively; KDIGO, Kidney Disease: Improving Global Outcomes; mild AKI, KDIGO stage 1 or 2 postoperatively; severe AKI, KDIGO stage 3 postoperatively. Each bar represents the interquartile range (25^th^–75^th^ percentile), ‘o’ represents the median; non-AKI, no AKI postoperatively; mild AKI, KDIGO stage 1 or 2 postoperatively; severe AKI, KDIGO stage 3 postoperatively. Lac: Lac_adm_, Lac_ca_, Lac_op_, Lac_rw_, Lac_cpb_, Lac_end_, Lac_po4h_, Lac_po12h_, and Lac_po24h_ represent the time points for measuring lactate: on admission to the operating room; prior to MHCA; 5 min after reinitiation of lower body perfusion; on completion of rewarming; at the end of CPB; at the end of surgery; and at 4 h, 12 h, and 24 h, respectively. *p < 0.05, between the severe-AKI and non-AKI cohorts; ^**+**^*p* < 0.05, compared between the severe-AKI and mild-AKI cohorts.

In all cohorts, lactate levels increased from admission to 24 h postoperatively, with a more significant increase during CPB, peaking at the end of surgery, then decreasing and approaching admission levels at 24 h postoperatively. Lactate levels at each time point were highest in the severe-AKI cohort, followed by the mild-AKI and non-AKI cohorts, with more significant differences at later time points during or after the surgical procedure (*p* < 0.05). Concerning the decline in postoperative lactate levels, these levels decreased rapidly in the non-AKI and mild-AKI cohorts, whereas the reduction in the severe AKI was less pronounced (Figure 1).

### 3.3 Diagnostic value of perioperative lactate levels for severe AKI

**Figure 2:**
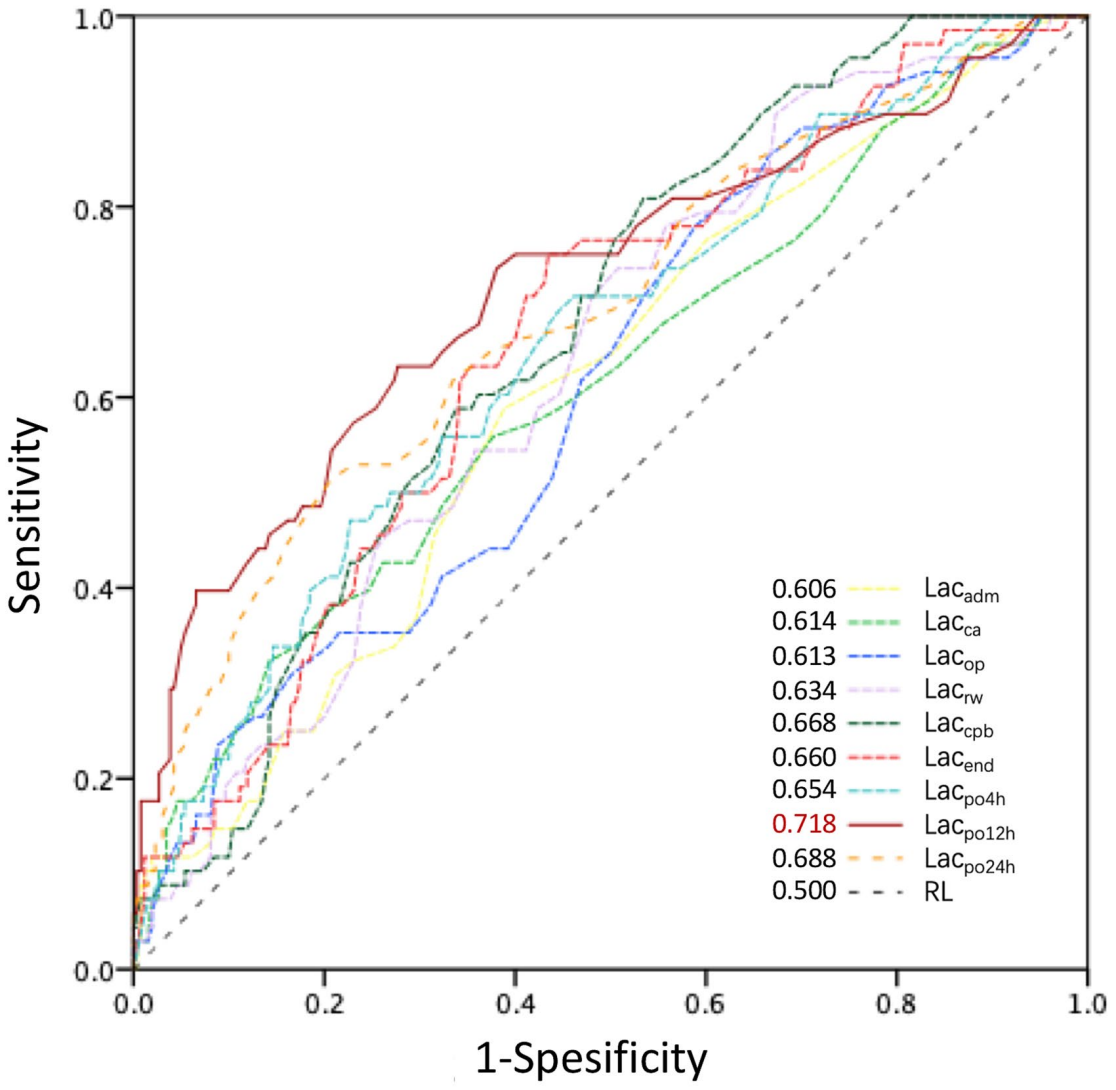
ROC curves for perioperative lactate levels influencing postoperative severe AKI. AKI, acute kidney injury; AUC, area under the curve; KDIGO, Kidney Disease: Improving Global Outcomes; severe AKI, KDIGO stage 3. Lac_adm_, Lac_ca_, Lac_op_, Lac_rw_, Lac_cpb_, Lac_end_, Lac_po4h_, Lac_po12h,_ and Lac_po24h_ represent lactate levels at the time of admission to the operating room; prior to MHCA; 5 min after reinitiation of lower body perfusion; on completion of rewarming; at the end of CPB; at the end of surgery; and at 4, 12, and 24 h postoperatively, respectively. The AUC of lactate levels at different time points is shown in the bottom-right corner. The most significant AUC value was 0.718 for Lac_po12h_.

The Mann–Whitney U test results showed significant differences in lactate levels between the severe-AKI and non-AKI cohorts at all time points (*p* < 0.05). Excluding the time points of admission to the operating room and before MHCA, significant differences in lactate levels were observed between the severe-AKI and non-AKI cohorts (*p* < 0.05; Figure 1; Supplementary Table E1). The predictive power for severe AKI was well-reflected by lactate levels at all time points, with particularly high predictive efficacy at 12 h postoperatively (Lac_po12h_, area under the curve: 0.718; cutoff value: 3.3 mmol/L; sensitivity: 63.2%; specificity: 72.3%; Figure 2).

### 3.4 Outcomes of univariate and multivariate logistic regression analysis

Considering the temporal sequence of other postoperative systemic complications, their association with AKI, and their influence on clinical outcomes, they were not included in the regression analysis. As postoperative sCr levels serve as a manifestation and diagnostic criterion for AKI, the creatinine level at 12 h postoperatively (sCr_po12h_) was not included in the logistic regression analysis despite significant differences (*p* < 0.05). Additionally, the cystatin C level and the eGFR, which are indicators of renal function, were excluded. Other perioperative indicators that showed statistically significant differences and passed collinearity diagnostics were included in the univariate analysis.

**Figure 3:**
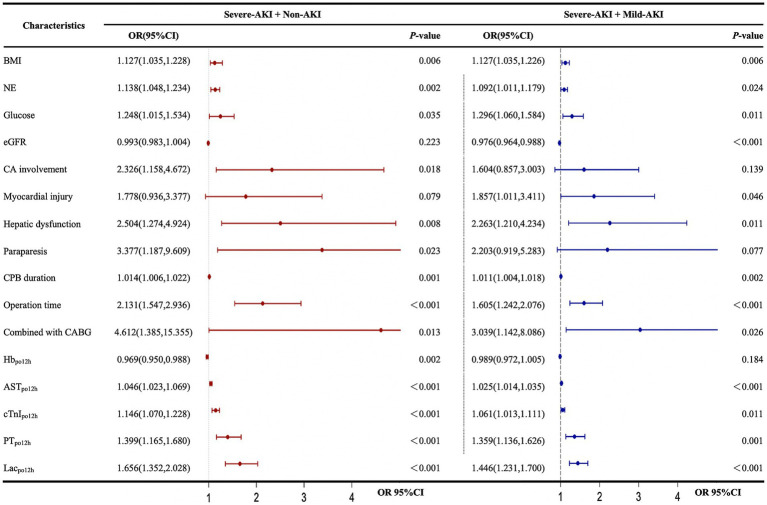
Univariate logistic regression analysis of factors influencing the development of postoperative severe AKI. AKI, acute kidney injury; AST, aspartate aminotransferase; BMI, body mass index; CA, coronary artery involvement; CABG, coronary artery bypass grafting; CI, confidence interval; CPB, cardiopulmonary bypass; cTnI, cardiac troponin I; eGFR, estimated glomerular filtration rate; Hb, hemoglobin; KDIGO, Kidney Disease: Improving Global Outcomes; Lac, lactate; NE, neutrophilic granulocyte; OR, odds ratio; PT, prothrombin time. The forest plot represents the ORs and 95% CIs of the corresponding characteristics: non-AKI, no AKI postoperatively; mild AKI, KDIGO stage 1 or 2 postoperatively; severe AKI, KDIGO stage 3 postoperatively; po12h, level at 12 h postoperatively.

Univariate analysis showed that body mass index, NE, glucose, coronary artery involvement, preoperative hepatic dysfunction, paraparesis, CPB duration, duration of surgery combined with coronary artery bypass grafting, hemoglobin_po12h_, AST_po12h_, cTnI_po12h_, PT_po12h_, and Lac_po12h_ were risk factors for severe AKI in the severe-AKI and non-AKI cohorts. In the severe-AKI and mild-AKI cohorts, except for coronary artery involvement (odds ratio [OR]: 1.604; 95% confidence interval [CI]: 0.857–3.003), preoperative paraparesis (OR: 2.203; 95% CI: 0.919–5.283), and hemoglobin_po12h_ (OR: 0.989; 95% CI: 0.972–1.005), the aforementioned indicators, preoperative myocardial injury (OR: 1.857, 95% CI: 1.011–3.411), and preoperative eGFR (OR: 0.976, 95% CI: 0.964–0.988) were identified as risk factors for severe AKI (Figure 3).

**Figure 4:**
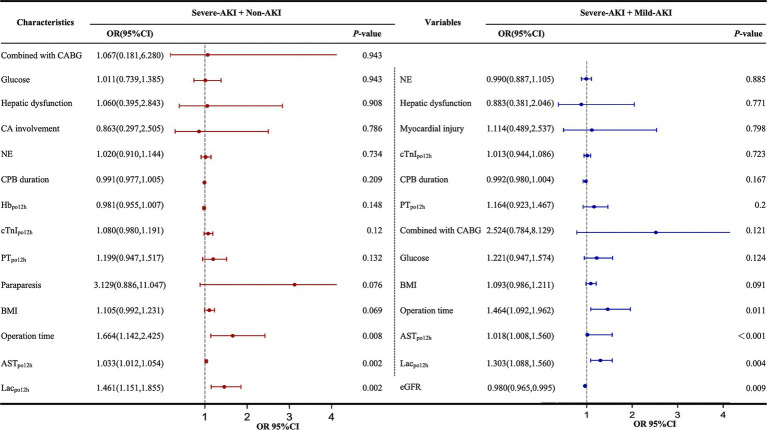
Multivariate logistic regression analysis of factors influencing severe AKI postoperatively. AKI, acute kidney injury; AST, aspartate aminotransferase; BMI, body mass index; CA, coronary artery involvement; CABG, coronary artery bypass grafting; CI, confidence interval; CPB, cardiopulmonary bypass; cTnI, cardiac troponin I; eGFR, estimated glomerular filtration rate; Hb, hemoglobin; KDIGO, Kidney Disease: Improving Global Outcomes; Lac, lactate; NE, neutrophilic granulocyte; ORs, odds ratios; PT, prothrombin time. The forest plot represents the ORs and 95% CIs of the corresponding characteristics; non-AKI, no AKI postoperatively; mild AKI, KDIGO stage 1 or 2 postoperatively; severe AKI, KDIGO stage 3 postoperatively; po12h, level at 12 h postoperatively.

Owing to the limited sample size, a stepwise backward method was employed to incorporate the independent risk factors identified from the univariate analysis into the multivariate analysis. Lac_po12h_, AST_po12h_, and operative duration were independent risk factors for severe AKI in all patients after multivariate adjustment. Additionally, a low preoperative eGFR (OR 0.98, 95% CI 0.965–0.995) was identified as an independent risk factor for severe AKI in the severe-AKI + mild-AKI cohort (Figure 4).

### 3.5 Subgroup analysis outcomes

**Figure 5:**
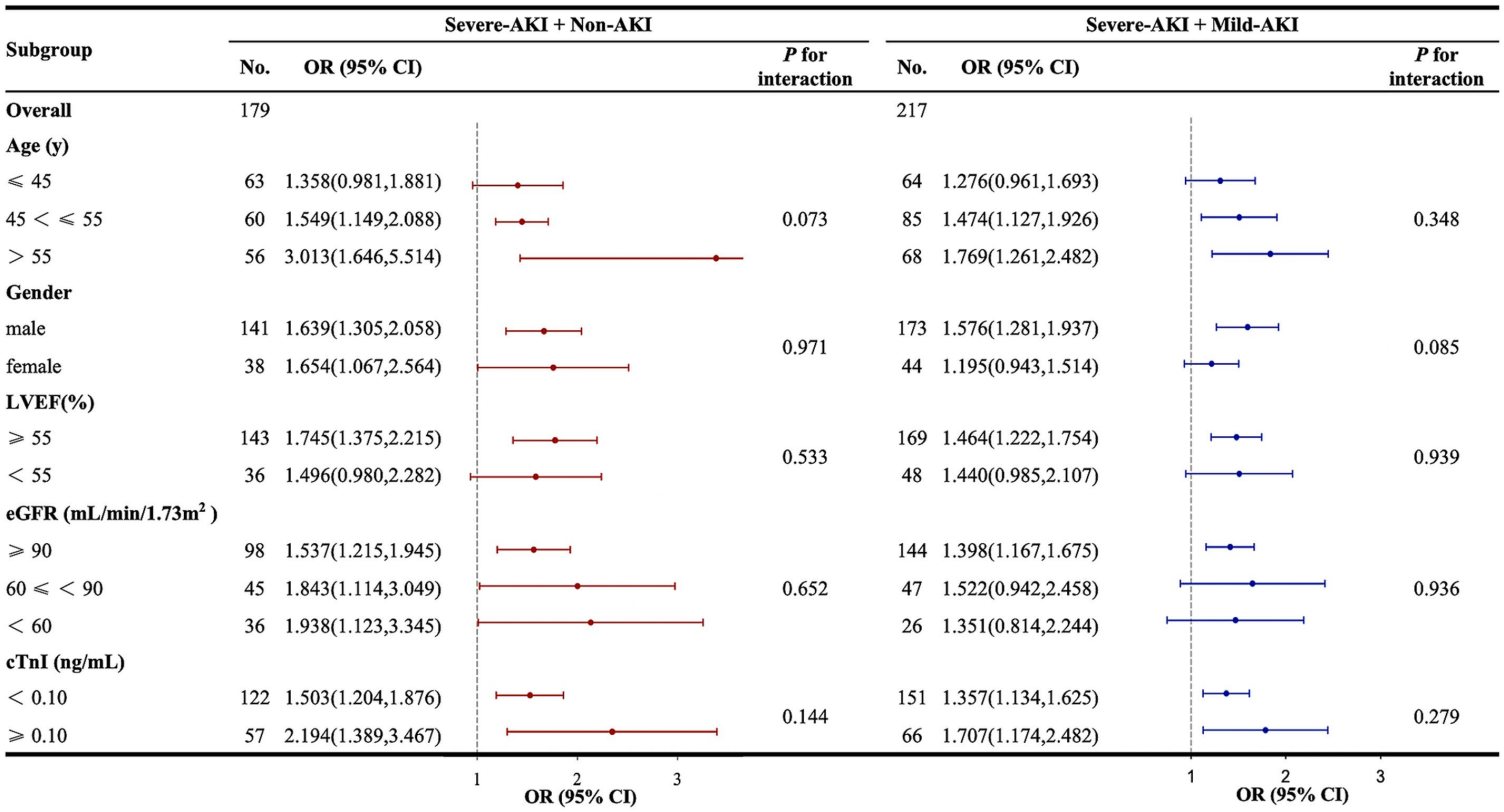
Subgroup and interaction analyses between Lac_po12h_ and postoperative severe AKI. AKI, acute kidney injury; CI, confidence interval; cTnI, cardiac troponin I; eGFR, estimated glomerular filtration rate; KDIGO, Kidney Disease: Improving Global Outcomes; Lac_po12h_, lactate levels at 12 h postoperatively; LVEF, left ventricular ejection fraction; ORs, odds ratios. The forest plot represents the ORs and 95% CIs for Lac_po12h_ to predict severe AKI in the corresponding subgroups: non-AKI, no AKI postoperatively; mild AKI, KDIGO stage 1 or 2 postoperatively; severe AKI, KDIGO stage 3 postoperatively.

In the severe-AKI + non-AKI cohort in relation to predicting severe AKI, Lac_po12h_ was more effective in patients aged >45 years than in those aged ≤45 years. Lac_po12h_ was also more effective in patients with an LVEF of ≥55% compared with those with an LVEF of <55%. However, there were no differences in the subgroups according to sex, eGFR, or cTnI levels. In the severe-AKI + mild-AKI cohort in relation to predicting severe AKI, the effect of Lac_po12h_ was better in subgroups of patients aged >45 years who were male, with an LVEF of ≥55%, and with eGFR of ≥90 mL/min/1.73 m^2^. No significant differences in the cTnI subgroup were observed. In all the subgroups, no interaction was observed between Lac_po12h_ and severe postoperative AKI (Figure 5).

### 3.6 Clinical outcomes

In the severe-AKI cohort, the incidence of cardiovascular system complications (45.6%), respiratory failure (51.5%), pulmonary infection (48.5%), stroke (13.2%), delirium (32.4%), and hepatic dysfunction (58.8%) was significantly higher (*p* < 0.05) than those in the other two cohorts. The 30-day postoperative mortality rates in the non-AKI, mild-AKI, and severe-AKI cohorts were 2.7, 2.7, and 48.5%, respectively, indicating significant differences (*p* < 0.001; Table 3). Lac_po12h_ levels in all patients who died within 30 days postoperatively were significantly higher than those in patients who survived (*p* < 0.05). The ROC curve analysis for perioperative lactate levels in relation to postoperative 30-day mortality indicated that lactate levels at four time points from the end of surgery to 24 h postoperatively all had a good predictive effect on postoperative mortality (Lac_end_, AUC = 0.639; Lac_po4h_, AUC = 0.723; Lac_po12h_, AUC = 0.780; Lac_po24h_, AUC = 0.811) (Supplementary Figure S2). It could be observed that higher lactate levels at time points further from the end of surgery had a more pronounced predictive effect on postoperative mortality.

## 4 Discussion

The relationship between lactate levels and AKI has previously been investigated (9–12). However, this is the first study to assess the predictive value of lactate levels at many perioperative time points for severe AKI following TAR combined with FET implantation in patients with ATAAD.

Perioperative lactate levels showed significant predictive value for severe postoperative AKI in these patients. Furthermore, Lac_po12h_ was an independent risk factor for severe AKI and exhibited reliable and robust predictive power (cutoff point: 3.3 mmol/L; sensitivity: 63.2%; specificity: 72.3%) and was also associated with postoperative survival. The incidence of complications and the mortality rate in patients with severe postoperative AKI were significantly higher, indicating that severe AKI led to extremely adverse clinical outcomes, as previously reported (13, 14).

Lactate, a byproduct of anaerobic metabolism, often causes tissue hypoxia and metabolic abnormalities. Impaired renal function affects lactate clearance (15). Therefore, dynamic changes in lactate levels can reflect the recovery of postoperative renal function, thereby serving as a predictive factor for severe postoperative AKI. The maximum intraoperative lactate level is known to be an independent risk factor for AKI following TAR combined with FET implantation (16). During MHCA in the TAR, the blood supply to the lower body is temporarily arrested, resulting in ischemia and hypoxia in the lower body organs for a certain period and the accumulation of lactate owing to metabolic processes (17). However, we considered that, while the peripheral blood supply was restored after perfusion reinitiation and these areas could function relatively well, high levels of lactate persisted owing to the effects of ongoing anesthesia, CPB, and surgical trauma. This implies that the predictive value of the intraoperative lactate level for AKI is not adequate. Once the dissection was corrected and surgical manipulation was concluded, the organs began to excrete the lactate that had accumulated intraoperatively. Similar to the changes we described, lactate levels rise intraoperatively, reach their highest level at the end of surgery, and then decrease after surgery. This might explain why Lac_po12h_, rather than other time points, was more significant and appropriate for predicting severe postoperative AKI in our study. Furthermore, according to this result, we considered that patients with relatively weaker kidney function might experience a slower decline and sustain higher postoperative lactate levels and that recovery of renal function within 12 h postoperatively might predict the short-term outcomes of kidney function and survival; however, this requires further evidence-based research.

Additionally, one study (18) reported a significant increase in the incidence of sepsis-induced AKI and renal replacement therapy with a blood lactate level of ≥4 mmol/L. Furthermore, *in vitro* and *in vivo* experiments showed that additional lactate administration directly promoted sepsis-induced AKI. Mechanistically, high lactate levels mediate the lactylation of mitochondrial fission 1 protein (Fis1) lysine 20 (Fis1 K20la), which promotes excessive mitochondrial fission and subsequently induces ATP depletion, mitochondrial reactive oxygen species overproduction, mitochondrial apoptosis, and exacerbates sepsis-induced AKI. Activation of certain pathways resulting from high lactate concentrations can cause cellular damage to organs. Elevated lactate levels reflect reduced kidney function and may indirectly cause renal tissue damage, strongly supporting the hypothesis that lactate plays an effective role in predicting postoperative AKI. In relation to our study results, when the Lac_po12h_ level exceeds 3.3 mmol/L, concerns relating to the development of severe AKI following TAR should be raised.

In cardiac surgery, preoperative renal dysfunction, which may suggest more extensive vascular disease, is a significant predictor of postoperative AKI (19). In our study, among patients with postoperative AKI, those with severe AKI presented with higher preoperative sCr and cystatin C levels and a lower eGFR. Multivariate analysis also indicated that a higher preoperative eGFR was a protective factor against severe AKI in patients with postoperative AKI. However, no significant difference in preoperative renal dysfunction was observed between the severe-AKI cohort and the other two cohorts. These indicators appear insufficient to evaluate preoperative acute renal impairment resulting from aortic dissection, necessitating the integration of additional information such as renal blood flow perfusion status and morphological function (20). Such information would facilitate an improved understanding of the effect of preoperative renal dysfunction on postoperative renal prognosis and guide clinical practice accordingly.

Studies investigating the correlation between preoperative cardiac function and postoperative AKI in cardiac surgery have also indicated that patients with preoperative cardiac dysfunction are more likely to develop postoperative AKI (21). This may be associated with inadequate blood volume during the peri-and postoperative cardiac surgery periods, leading to reduced renal perfusion and increased susceptibility to ischemia and hypoxia, thereby resulting in kidney damage (22). A large-sample study showed that biomarkers related to cardiac function could predict cardiovascular complications post-cardiac surgery and AKI (23). However, patients who underwent aortic surgery were excluded from that study. Considering the cardiorenal axis, we attempted to analyze the association between cardiac function and postoperative AKI. We analyzed cardiac function indicators including LVEF, cTnI, and N-terminal pro-B-type natriuretic peptide, and our findings indicated that preoperative myocardial injury (cTnI ≥0.1) and cTnI_po12h_ were both risk factors for postoperative severe AKI. However, owing to the influence of other factors, they could not predict severe AKI independently and did not influence the predictive value of Lac_po12h,_ according to the subgroup analysis results.

Prolonged CPB and operation duration are significant factors associated with the development of postoperative AKI (24). The spectrum of CPB-induced pathophysiological changes includes a systemic inflammatory response, changes in renal vasomotor tone, destruction of red blood cells, pigment nephropathy, loss of pulsatile flow, activation of complement and coagulation pathways, and generation of microemboli (fibrin, platelet aggregates, cellular debris, fat, and air). These factors are associated with complex yet unconfirmed mechanisms underlying the development of AKI. Prolonged CPB inevitably amplifies these effects, thereby increasing the risk of AKI (25). In addition, the operative time has been identified as a significant contributor to postoperative AKI. The complexity and technical requirements of the TAR procedure, coupled with the necessity for CPB support, inevitably result in a prolonged operation duration, which can lead to more complex physiological stress responses, including renal hypoperfusion, ischemia–reperfusion injury, and high lactate levels, thereby increasing the risk of postoperative AKI (26). In the present study, both conditions were associated with severe AKI. However, only operation time independently predicted severe postoperative AKI, which might be due to a bias caused by the small sample size. In terms of determining a method to mitigate the risks associated with CPB, the findings in one study have been encouraging (27). A goal-directed perfusion strategy during CPB effectively reduced Acute Kidney Injury Network stage 1 AKI. Further studies are required to define perfusion interventions that may reduce the severity of renal injury.

The level of AST_po12h_ was also an independent risk factor for severe AKI in our study, but we considered that the relationship between AST and renal function prognosis might not be direct. A literature review found that postoperative AKI is a common complication of liver transplantation. Hepatic ischemia–reperfusion may be an important factor in the occurrence of AKI after liver transplantation (28). Patients with AKI and with increased AST and gamma-glutamyl transferase levels require special attention to improve their prognosis. Hepatic ischemia–reperfusion owing to CPB during TAR surgery affects liver function and leads to postoperative abnormalities. Increased AST levels may indicate hepatic dysfunction, which can affect drug metabolism and detoxification, thereby affecting the recovery of kidney function. In addition, the liver and kidneys have a synergistic effect on the metabolism and excretion of lactate and other wastes. Therefore, liver insufficiency might increase the burden on the kidneys, affecting renal function and potentially explaining the indirect predictive effect of postoperative AST levels on AKI. Given this synergistic effect, we hypothesized that the burden of lactate excretion on the kidneys might increase in patients with hepatic dysfunction. If renal function is compromised, lactate levels increase significantly. Consequently, we investigated whether lactate levels could better predict AKI in patients with hepatic dysfunction. We stratified the subgroups based on postoperative hepatic dysfunction and found no interaction (*p* = 0.415 in the severe-AKI + non-AKI cohort; *p* = 0.752 in the severe-AKI + mild-AKI cohort) between high Lac_po12h_ levels and hepatic dysfunction in predicting severe AKI.

## 5 Limitations

This study has some limitations. The study design was a time-based comparison, with potential bias related to time. The majority of patients were from Northwest China, which might have introduced population bias. The data were obtained from a small-sample single-center database, potentially introducing data bias. Finally, long-term outcomes were not collected; therefore, follow-ups should be conducted for all such patients.

## 6 Conclusion

Patients undergoing TAR combined with FET implantation for ATAAD were at a higher risk of severe postoperative AKI associated with a poor prognosis. High perioperative lactate levels were significantly associated with severe AKI. Furthermore, Lac_po12h_ was identified as a reliable and effective predictor of severe postoperative AKI, with the potential to enhance the capacity of existing AKI prediction models and facilitate clinical strategies.

## Data Availability

Publicly available datasets were analyzed in this study. This data can be found at: Electronic Medical Record System of Xijing Hospital.
