# Supplementary material for: High perioperative lactate levels as a potential predictor for severe acute kidney injury following aortic arch surgery
Source: Front Med (Lausanne). 2025 Jan 6;11:1495502. doi: 10.3389/fmed.2024.1495502 (PMC11743281; doi:10.3389/fmed.2024.1495502)
Supplement: Supplementary file 1 [file Table_1.DOCX]

**TABLE 1 Patient perioperative lactate levels by different AKI severity**

| **Characteristic** | **ALL(n=328)** | **Non-AKI(n=111)** | **Mild-AKI (n=149)** | **Severe-AKI (n=68)** | ***P*-value** | |
| --- | --- | --- | --- | --- | --- | --- |
|  |  |  |  |  | **Severe-AKI vs Non** | **Severe-AKI vs Mild** |
| Lac_adm_ | 1.20(0.90，1.87) | 1.10(0.80，1.60) | 1.20(0.90，2.00) | 1.50(1.10，2.15) | 0.001 | 0.077 |
| Lac_ca_ | 1.70(1.20，2.40) | 1.50(1.10，2.10) | 1.80(1.40，2.45) | 2.00(1.40，2.97) | ＜0.001 | 0.076 |
| Lac_op_ | 4.65(3.70，5.60) | 4.30(3.50，5.50) | 4.70(3.70，5.55) | 4.80(4.32，6.27) | 0.001 | 0.038 |
| Lac_rw_ | 4.65(3.50，5.97) | 4.40(3.10，5.60) | 4.70(3.50，5.85) | 5.30(4.30，6.45) | ＜0.001 | 0.010 |
| Lac_cpb_ | 5.70(4.20，7.57) | 5.00(3.30，6.80) | 5.90(4.15，7.40) | 7.05(5.30，8.40) | ＜0.001 | 0.001 |
| Lac_end_ | 6.35(3.80，9.10) | 5.50(3.20，8.50) | 6.10(3.70，8.65) | 7.95(6.17，9.80) | ＜0.001 | ＜0.001 |
| Lac_po4h_ | 4.30(3.00，7.10) | 4.10(2.60，6.20) | 4.10(2.90，6.50) | 5.95(3.62，9.07) | ＜0.001 | 0.001 |
| Lac_po12h_ | 2.60(1.80，4.00) | 2.40(1.60，3.50) | 2.50(1.80，3.65) | 3.85(2.50，5.80) | ＜0.001 | ＜0.001 |
| Lac_po24h_ | 1.70(1.20，2.40) | 1.50(1.10，1.90) | 1.70(1.20，2.40) | 2.40(1.50，3.57) | ＜0.001 | ＜0.001 |

a: Abbreviations: Lac_adm_、Lac_ca_、Lac_op_、Lac_rw_、Lac_cpb_、Lac_end_、Lac_po4h_、Lac_po12h_ and Lac_po24h_ represent lactate levels at the time of admission to operation room, prior to MHCA, 5 minutes after re-initiation of lower body perfusion, completion of rewarming, the end of cardiopulmonary bypass, the end of surgery, and at postoperative 4th, 12th, and 24th hour, respectively.

b: Non-AKI represented no occurrence of any AKI following surgery. Mild-AKI is KDIGO stage 1 or 2 following surgery. Severe-AKI is KDIGO stage 3 following surgery.

c: All normally distributed continuous variables were described with means (±standard deviation [SD]) and compared across groups with Student t-test. Median (interquartile range [IQR]) and the Wilcoxon rank sum test were used for non-normally distributed continuous variables. Categorical variables were described with frequencies (%) and compared with chi-square test or Fisher’s exact test.

d: Severe-AKI vs Non: Patients with severe-AKI (KDIGO stage 3) compared with patients without AKI.

e: Severe-AKI vs Mild: Patients with severe-AKI (KDIGO stage 3) compared with patients with mild-AKI (KDIGO stage 1or 2).
