# Supplementary figures and images for: High perioperative lactate levels as a potential predictor for severe acute kidney injury following aortic arch surgery
Source: Front Med (Lausanne). 2025 Jan 6;11:1495502. doi: 10.3389/fmed.2024.1495502 (PMC11743281; doi:10.3389/fmed.2024.1495502)

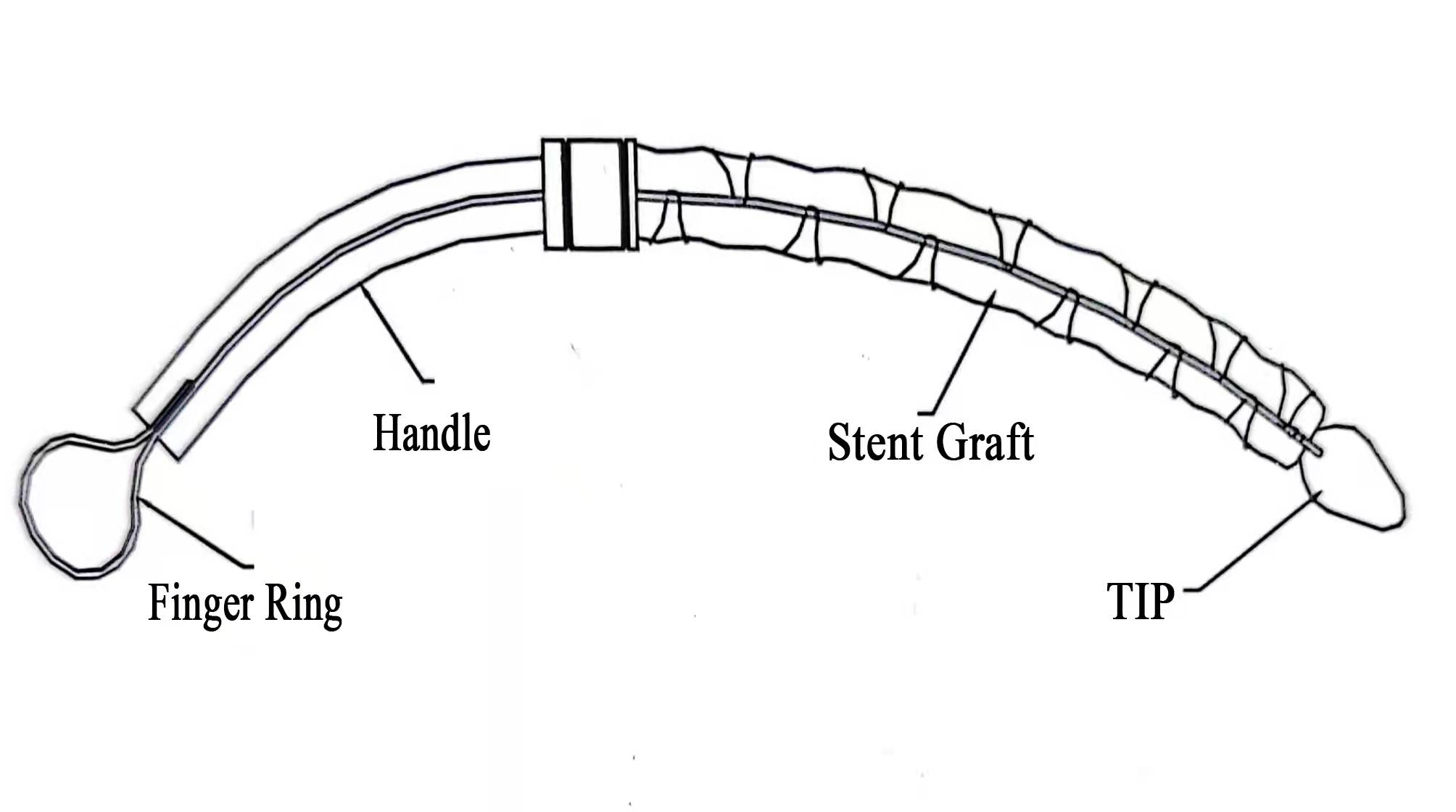

Supplement: Supplementary file 2 [file Image_1.TIF]

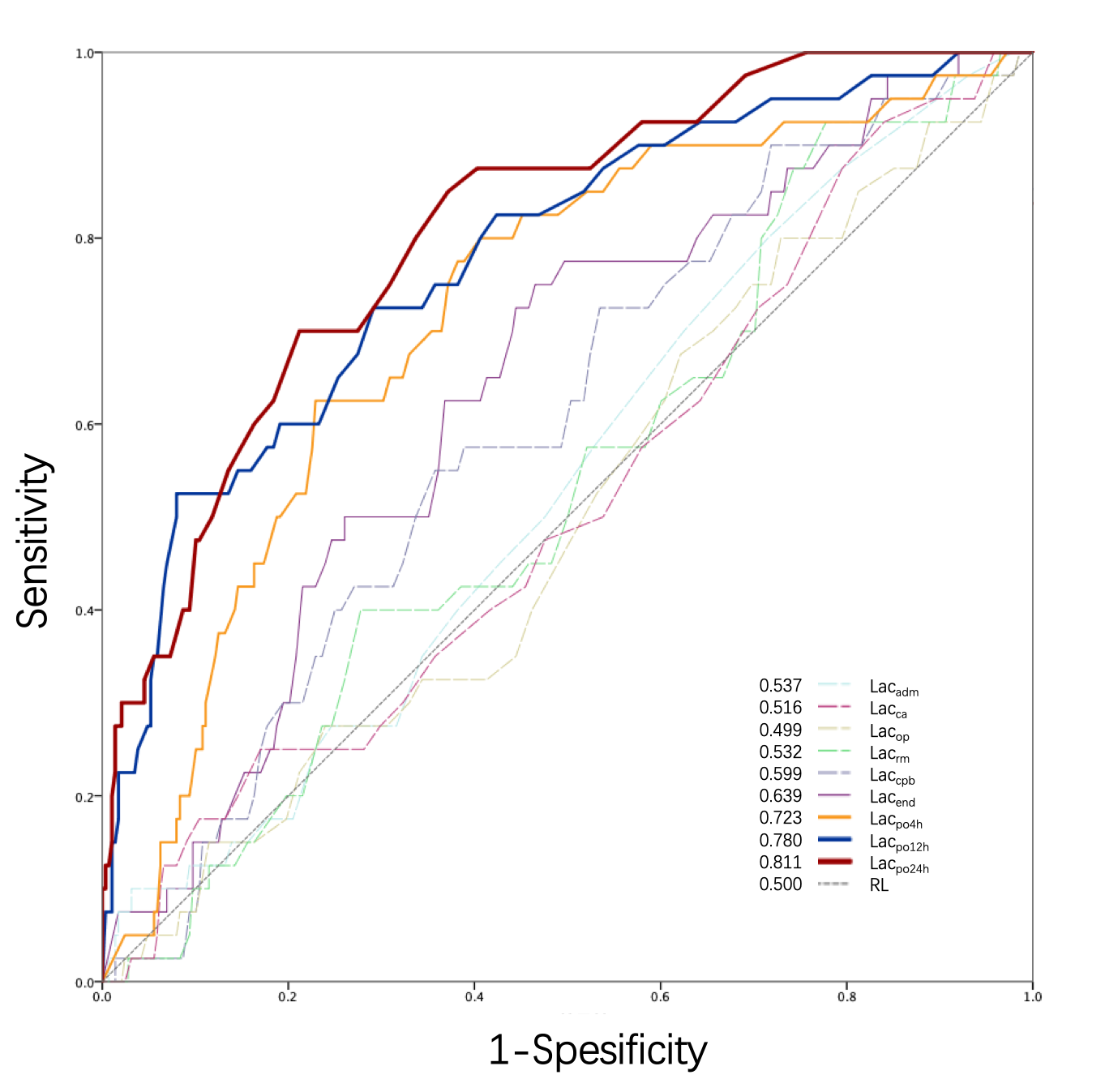

Supplement: Supplementary file 3 [file Image_2.TIF]
